# The Association Between Hypertensive Disorders in Pregnancy and the Risk of Developing Chronic Hypertension

**DOI:** 10.3389/fcvm.2022.897771

**Published:** 2022-07-07

**Authors:** Jiahao Xu, Ting Li, Yixiao Wang, Lu Xue, Zhijing Miao, Wei Long, Kaipeng Xie, Chen Hu, Hongjuan Ding

**Affiliations:** ^1^Department of Obstetrics and Gynecology, Nanjing Maternity and Child Health Care Hospital, Women’s Hospital of Nanjing Medical University, Nanjing, China; ^2^Department of Public Health, Nanjing Maternity and Child Health Care Hospital, Women’s Hospital of Nanjing Medical University, Nanjing, China; ^3^Department of Women Health Care, Nanjing Maternity and Child Health Care Hospital, Women’s Hospital of Nanjing Medical University, Nanjing, China

**Keywords:** hypertensive disorders in pregnancy (HDP), preeclampsia (PE), gestational hypertension (GH), hypertension, pooled odds ratios (ORs), confidence intervals (CIs), systematic review, meta-analysis

## Abstract

**Objective:**

This meta-analysis comprehensively evaluated the association between hypertensive disorders in pregnancy (HDP) and the risk of developing chronic hypertension and the associations between specific types of HDP, including preeclampsia (PE) and gestational hypertension (GH), and the risk of developing chronic hypertension.

**Design:**

Systematic review and meta-analysis.

**Data Sources:**

The PubMed, Embase and Cochrane Library databases were searched from inception to August 20, 2021.

**Methods:**

Depending on heterogeneity, the combined odds ratio (OR) of the 95% confidence interval (CI) was obtained with a random-effects or fixed-effects model. We used meta-regression analysis to explore the sources of heterogeneity. We analyzed the OR value after adjusting for age and BMI at recruitment, prepregnancy BMI, age at first delivery, and other factors. Additionally, we evaluated the results of the subgroup analysis by the year of publication (< 2016, ≥ 2016), study design, sample size (< 500, ≥ 500), region (North and South America, Europe, and other regions) and NOS score (< 7, ≥ 7).

**Results:**

Our systematic review and meta-analysis comprehensively explored the relationships between HDP, GH, and PE and chronic hypertension. Twenty-one articles that included 634,293 patients were included. The results of this systematic review and meta-analysis suggested that women with a history of HDP are almost 3.6 times more likely to develop chronic hypertension than those without a history of HDP, women with a history of GH are almost 6.2 times more likely to develop chronic hypertension than those without a history of GH, and women with a history of PE are almost 3.2 times more likely to develop chronic hypertension than those without a history of PE. In addition, we further calculated the probability of developing chronic hypertension among patients with HDP or PE after adjusting for age and BMI at recruitment, prepregnancy BMI, age at first delivery, and other factors. The results suggested that women with a history of HDP are almost 2.47 times more likely to develop chronic hypertension than those without a history of HDP and that women with a history of PE are almost 3.78 times more likely to develop chronic hypertension than those without a history of PE. People in Asian countries are more likely to develop chronic hypertension after HDP or PE, while American people are not at high relative risk.

**Conclusion:**

These findings suggest that HDP, GH, and PE increase the likelihood of developing chronic hypertension. After adjustment for age and BMI at recruitment, prepregnancy BMI, age at first delivery, and other factors, patients with HDP or PE were still more likely to develop chronic hypertension. HDP may be a risk factor for chronic hypertension, independent of other risk factors. GH and PE, as types of HDP, may also be risk factors for chronic hypertension.

**Systematic Review Registration:**

[www.ClinicalTrials.gov], identifier [CRD42021238599].

## Introduction

Hypertension is one of the most common conditions that occur during pregnancy and the main cause of maternal death (1). Ten percent of pregnancies are affected by hypertension, especially those of primiparas. Hypertensive disorders in pregnancy (HDP) include a series of diseases classified as preeclampsia, eclampsia, gestational hypertension, pregnancy complicated with chronic hypertension and preeclampsia superimposed on chronic hypertension (2). Their definitions are shown in Table 1. HDP remains one of the leading causes of maternal and fetal disease incidence and mortality worldwide. Moreover, HDP is closely related to the patient’s future health. A study found that women with prepregnancy hypertension and those with both HDP and prepregnancy hypertension had an increased risk of kidney disease 5 years after delivery (3). HDP increases the risk of future cardiovascular events and has been included in the guidelines for the risk assessment and prevention of stroke and cardiovascular disease (CVD) in women (4, 5). Recent evidence indicates that the incidence rate of HDP has increased over the past 30 years, suggesting that HDP, a sex-specific CVD risk factor, may become more important in the coming years (6, 7). A history of gestational hypertension/preeclampsia is related to subclinical atherosclerosis (increased carotid intima-media thickness (IMT) and plaque) (8). Pregnancy-induced hypertension is even hereditary, affecting the cardiovascular health of offspring (9).

**TABLE 1:** Definition of HDPs.

Type of HDPs	Definition
Gestational hypertension	Hypertension occurring after 20 weeks of pregnancy, systolic blood pressure ≥ 140 mmHg and (or) diastolic blood pressure ≥ 90 mmHg, and a return to normal blood pressure within 12 weeks after delivery; urinary protein (–); the diagnosis can be made after delivery.
Preeclampsia	Systolic blood pressure ≥ 140 mmHg and (or) diastolic blood pressure ≥ 90 mmHg after 20 weeks of pregnancy, accompanied by urinary protein ≥ 0.3 g/24 h, or random urinary protein (+) Or without proteinuria, but combined with any of the following: • Thrombocytopenia (platelets < 100) × 10^9/^L) • Liver function impairment (serum transaminase level is more than twice the normal value) • Renal function impairment (serum creatinine level > 1.1 mg/dl or more than twice the normal value) • Pulmonary edema • New central nervous system abnormalities or visual impairment
Eclampsia	Convulsions that cannot be explained by other reasons occurring on the basis of preeclampsia.
Preeclampsia superimposed on chronic hypertension	There was no proteinuria before pregnancy, and proteinuria was present after 20 weeks of pregnancy in women with chronic hypertension; or proteinuria was present before pregnancy, and proteinuria increased significantly after pregnancy; or blood pressure rises further; or thrombocytopenia < 100 × l0^9/^L; or other serious manifestations such as liver and kidney function damage, pulmonary edema, nervous system abnormalities, or visual impairment.
Pregnancy complicated with chronic hypertension	Systolic blood pressure ≥ 140 mmHg and (or) diastolic blood pressure ≥ 90 mmHg before 20 weeks of pregnancy (excluding trophoblastic diseases), and there was no significant aggravation during pregnancy; or hypertension was first diagnosed after 20 weeks of pregnancy and continued beyond 12 weeks postpartum.

Studies have shown that women with preeclampsia have a higher risk of developing chronic hypertension. Indeed, comprehensive data show that 20% of women with eclampsia develop hypertension within 15 years (10). However, the risk varies depending on the population studied and the criteria used for diagnosis. According to a study, the risk of hypertension in Sweden 5–12 years after pregnancy is approximately 40% (11, 12). Three other studies reached similar conclusions (13–15). The correlation between HDP and chronic hypertension fluctuates greatly. The results were different depending on the region and follow-up years. There are many other confounding factors, such as race or country; studies have shown that African women with a history of pregnancy-induced hypertension, followed by Hispanic and Asian women, have the highest risk of future high blood pressure. Moreover, individuals with normal blood pressure showed better health-related quality of life than patients with hypertension. Although systemic hypertension has almost always been considered a clinically asymptomatic disease, it can impair the quality of life of patients (16, 17). Therefore, the early prevention of hypertension is necessary. If the association between gestational hypertension and chronic hypertension can be identified, the early prevention and treatment of HDP will greatly benefit the long-term health of patients.

This systematic review and meta-analysis assessed recent reports to explore the association between HDP and chronic hypertension and evaluate the associations between specific types of HDP, including preeclampsia (PE), and gestational hypertension (GH), and the risk of developing chronic hypertension. We analyzed both crude and adjusted OR values to better determine the relationships between the variables and the stability of the results. We also conducted subgroup analysis by country and year to analyze the relationship between HDP and chronic hypertension.

## Methods

This systematic review was conducted according to the Preferred Reporting Items for Systematic Reviews and Meta-Analyses (PRISMA) guidelines (18).

### Protocol, Eligibility Criteria, Information Sources, and Search Strategy

This review was based on a prior design recommended by a systematic review and meta-analysis. The PubMed, EMBASE, and Cochrane Library databases were searched electronically in August 2021 using a combination of terms, keywords and word variants related to the medical subject headings (MeSH) “hypertension, pregnancy,” “preeclampsia,” “eclampsia” and “hypertension.” We used Endnote x9 to remove duplicate articles and then browsed the titles and summaries to exclude unrelated articles. Reviews, meta-analyses, articles lacking relevant data, letters and abstracts were excluded. There were no time or language restrictions. The reference lists of relevant articles and comments were manually searched for additional reports. The study was registered in the Prospero database (Registration number: CRD42021238599).

### Study Selection, Data Collection, and Data Items

The main outcome was the incidence rate of chronic hypertension in patients with HDP or with the specific types PE and GH. We included case–control studies and cohort studies that provided data on how many patients developed hypertension several years after delivery. The research period of the different studies varied: the span was large, and the time period ranged from 1 to 30 years. Hypertension was defined as a systolic blood pressure (SBP) ≥ 140 mmHg and/or a diastolic blood pressure (DBP) ≥ 90 mmHg occurring more than once in a clinical environment. The use of antihypertensive drugs and lower thresholds for defining hypertension were also included in the diagnostic criteria. When data were available, only patients affected by HDP, PE, and GH were considered in the analysis. We excluded studies in which chronic hypertension was present before pregnancy or before 20 weeks of gestation. If a study included patients with chronic hypertension, we considered only the articles that provided the number of patients with chronic hypertension. In addition, we did not include articles about the incidence rate of postpartum hypertension within 1 year of delivery.

Two researchers, Xu and Wang, independently performed all abstract screenings. The two researchers retrieved and independently evaluated the full texts of potentially eligible studies. Any inconsistencies or differences were discussed with a third reviewer, and a consensus was reached. Several articles were translated into languages other than English to determine whether they were suitable for inclusion. The reviewers extracted data on the study characteristics and results, especially the author, year, location, study type, population size, and reported results. If multiple studies with the same endpoint were published for the same cohort, the report containing the most comprehensive population information was used to avoid population overlap.

### Risk of Bias and Study Quality

The quality of the included studies was assessed using the Newcastle–Ottawa Scale (NOS) for cohort and case–control studies, which was developed by Schokker et al. to assess the quality of non-randomized studies (19). With this protocol, the maximum score for each study was 9. Studies with a score ≥ 7 were considered high-quality articles. The two authors independently reviewed each study and determined whether it was eligible for inclusion in our meta-analysis. If there were any differences, the third author joined the discussion. Since the NOS could not be used to fully evaluate the potential confounding factors in the study analysis, information on which confounding factors were considered in each study was further extracted. Publication bias was assessed by a funnel plot using Begg’s and Egger’s tests (20). Subgroup analysis by publication year (< 2016, ≥ 2016), study design, location, sample size (< 500, ≥ 500) and NOS score (< 7, ≥ 7) was performed to further evaluate the associations between HDP, PE, and GH and chronic hypertension.

### Statistical Analysis

We constructed forest plots to obtain pooled ORs and 95% CIs. We applied a fixed-effects model to calculate the combined effect estimate if I_2_ ≥ 50%. Otherwise, we used a random-effects model. Sensitivity analysis was used to explore the robustness of the included literature. Publication bias was assessed by funnel plots and linear regression equations. If the funnel plot was obviously asymmetric, we further used the trim-and-fill method to adjust the data. In addition, meta-regression analysis was performed based on the publication year, NOS score, status, sample size, and study design to explore the sources of heterogeneity. All analyses were conducted *via* R version 3.6. The critical value for statistical significance was set as *P* < 0.05.

## Results

### Study Selection

**FIGURE 1:**
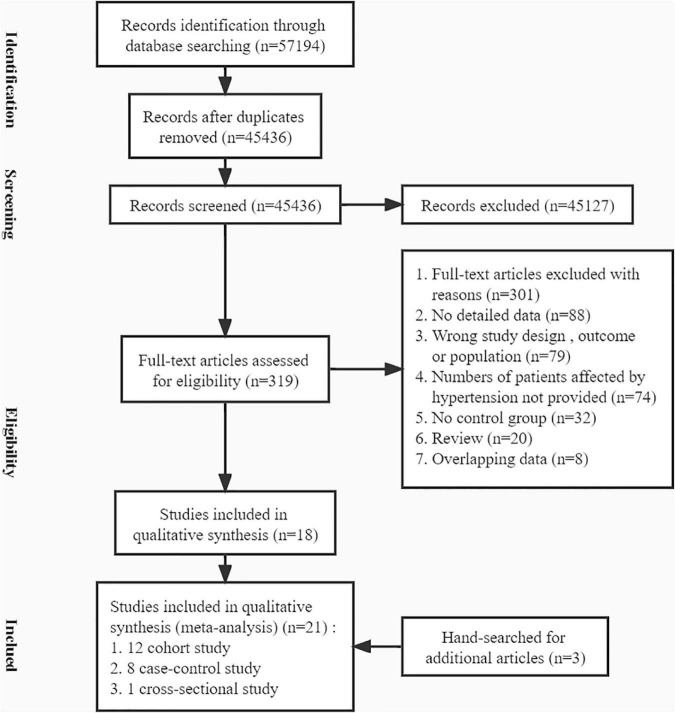
A flow chart of the screening of studies on HDP/GH/PE and chronic hypertension.

To obtain relevant literature, we searched the PubMed, Embase and Cochrane Library databases from inception to August 20, 2021. A total of 57,194 studies were obtained (Figure 1). After removing duplicate articles, 45,436 articles remained. Then, we culled articles that were unrelated and lacked data by scanning the titles, abstracts, and full texts. In addition, three studies that were retrieved from the reference lists of previous relevant articles were included. Ultimately, 21 studies met all eligibility criteria (21–41).

### Study Characteristics

The 21 studies included in this systematic review and meta-analysis varied in study design, year of publication, NOS score, country, and sample size. All studies were observational; 12 were described as cohort studies, eight as case–control studies and one as a cross-sectional study. The publication dates of these articles ranged from 2000 to 2021. Among these articles, the study areas included Europe for seven studies, North and South America for nine studies, and other regions for five studies. The smallest sample size was 28 (38), and the largest sample size was 331,707 (35). Eleven studies researched HDP, 3 researched GH, and 13 researched PE. There were five studies that included more than one disease. The research characteristics are summarized in Table 2.

**TABLE 2:** Summary characteristics of the 21 studies included in the systematic review of pregnancy complications.

Articles	Publication years	Country	Study design	Included population	Sample size	NOS score
Garrido-Gimenez et al. (21)	2020	France	Cohort study	PE	79	6
Moreira et al. (22)	2009	Brazil	Cross-sectional study	HDP	1,141	7
Drost et al. (23)	2011	Netherlands	Cohort study	PE	874	7
Watanabe et al. (24)	2020	Japan	Case–control study	HDP/GH/PE/OTHER	245	7
Nordén Lindeberg and Hanson (25)	2000	Sweden	Cohort study	HDP	115	8
Shammas and Maayah (26)	2000	Jordan	Case–control study	GH/PE	180	6
Garovic et al. (27)	2010	America	Case–control study	HDP	5,796	6
Wilson et al. (28)	2003	Britain	Cohort study	GH/PE	1,865	8
Mito et al. (29)	2018	Japan	Cohort study	HDP	796	7
Garovic et al. (30)	2020	America	Cohort study	HDP/PE	3,283	7
Edlow et al. (31)	2009	America	Case–control study	PE	248	7
Honigberg et al. (32)	2019	Britain	Cohort study	HDP	277,011	7
Marín et al. (33)	2000	Spain	Cohort study	HDP	463	8
Kuo et al. (34)	2018	China	Cohort study	PE/Eclampsia	7,050	7
Gastrich et al. (35)	2020	America	Case–control study	PE	331,707	6
White et al. (36)	2016	America	Cohort study	PE	112	8
Qasim et al. (37)	2016	Pakistan	Case–control study	HDP	527	7
Ghossein-Doha et al. (38)	2014	Netherlands	Cohort study	PE	28	6
Ehrenthal et al. (39)	2014	America	Case–control study	HDP	82	6
Shahul et al. (40)	2018	America	Case–control study	HDP/PE	137	6
Martelly et al. (41)	2021	America	Cohort study	PE	55	7

### Total Pooled Effect

**FIGURE 2:**
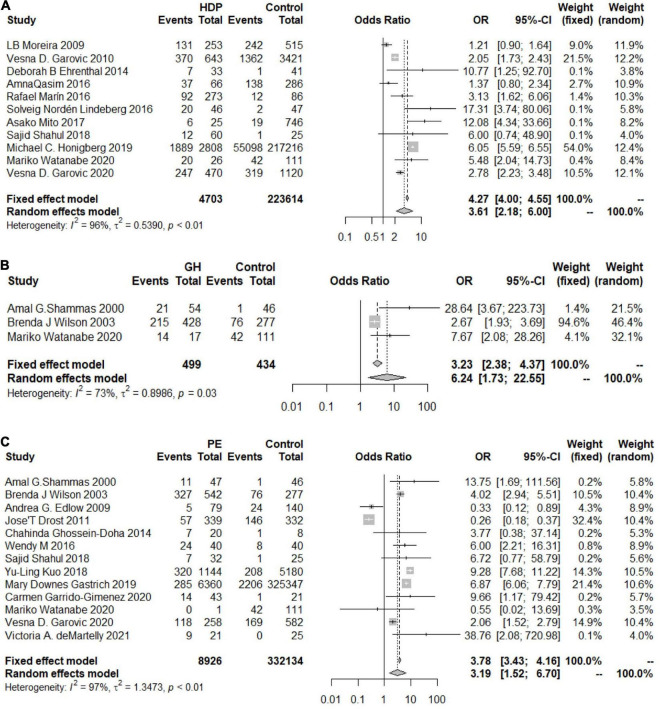
Forest plots of the risk of developing chronic hypertension. **(A)** Forest plots of the risk of developing chronic hypertension in the HDP group; **(B)** forest plots of the risk of developing chronic hypertension in the GH group; **(C)** forest plots of the risk of developing chronic hypertension in the PE group.

As shown in Figure 2A, the heterogeneity among the eligible articles about HDP was *I*^2^ = 96% (*P*< 0.01), so we chose to use a random-effects model. The overall combined effect showed that HDP patients had a higher risk of developing chronic hypertension than healthy controls (OR 3.61, 95% CI 2.18–6.00). We also calculated the GH and PE results and chose to use random-effects models (*I*_*GH*_^2^ = 73%, *P* = *0.03, I_*PE*_*^2^ = 97%, *P* < 0.01). Women with GH or PE were at higher risk of developing chronic hypertension than healthy controls (OR_GH_ 6.24, 95% CI 1.73–22.55, OR_PE_ 3.19, 95% CI 1.52–6.70) (Figures 2B,C).

Some articles reported adjusted OR values for age and BMI at recruitment, prepregnancy BMI, age at first delivery and other factors. We further evaluated the associations between HDP, GH, and PE and chronic hypertension based on the adjusted OR values.

**FIGURE 3:**
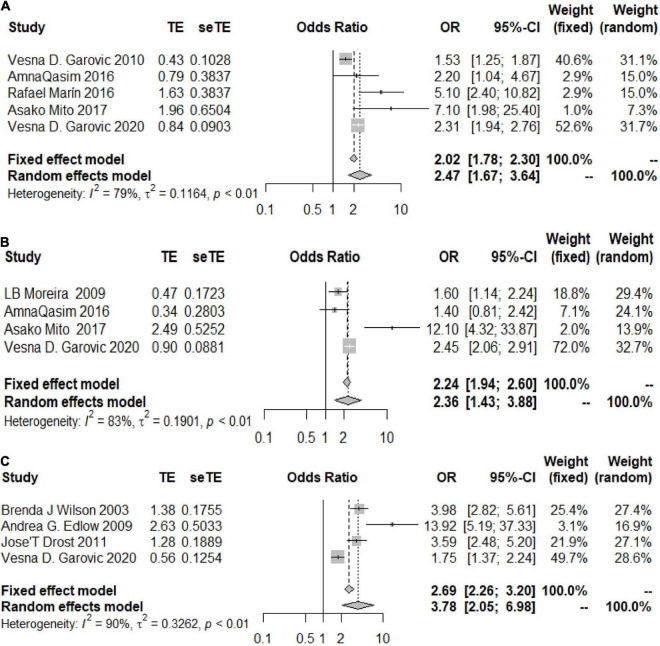
Forest plots of the risk of developing chronic hypertension in the adjusted group. **(A)** Forest plots of the risk of developing chronic hypertension in the HDP-adjusted group; **(B)** forest plots of the risk of developing chronic hypertension in the HDP-unadjusted group; **(C)** forest plots of the risk of developing chronic hypertension in the PE-adjusted group.

The heterogeneity among the articles about HDP with adjusted OR values was 79% (OR 2.47, 95% CI 1.67–3.64) (Figure 3A), and the heterogeneity among those with unadjusted OR values was 83% (OR 2.36, 95% CI 1.43–3.88) (Figure 3B). The two results were similar, showing that patients with HDP are at higher risk of developing chronic hypertension than healthy controls. The same trend in the risk of chronic hypertension was observed in the PE group, and the OR values were adjusted (*I*^2^ = 90%, OR = 3.78, 95% CI 2.05–6.98) (Figure 3C).

### Publication Bias, Sensitivity Analysis and Risk Analysis

**FIGURE 4:**
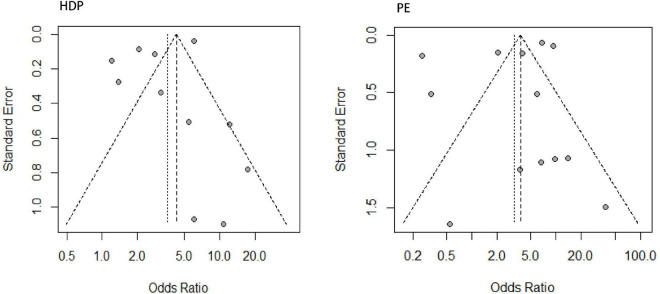
Funnel plot of the included studies for the HDP and PE groups.

**FIGURE 5:**
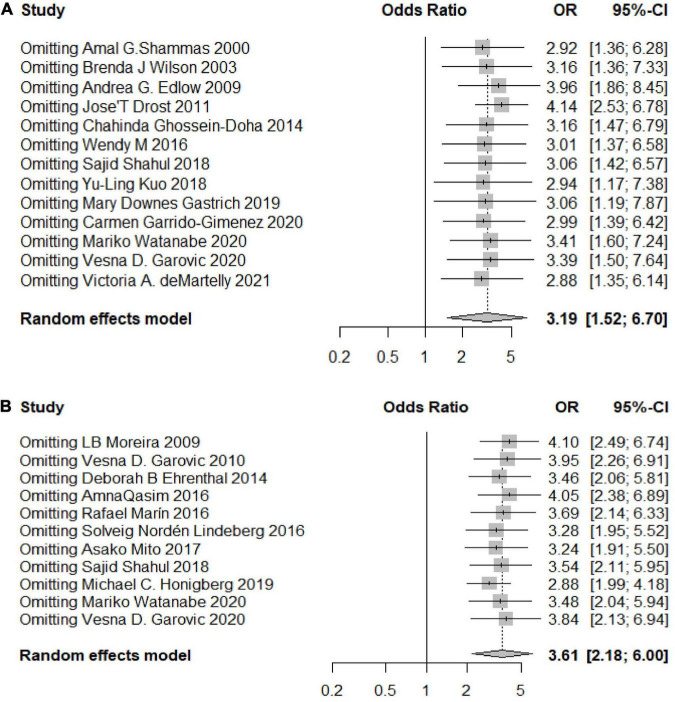
Sensitivity analyses of the included studies. **(A)** Sensitivity analysis of the included studies for the HDP group; **(B)** sensitivity analysis of the included studies for the PE group.

Through linear regression and funnel plots, we found that studies on HDP (*P* = 0.4639) and PE (*P* = 0.5380) had no publication bias (Figure 4). Figure 5A shows that when omitting one of these studies (22), the sensitivity analysis of the HDP group showed an OR of 4.10 (95% CI 2.49–6.74), which was nearly the same outcome as the total pooled effect (OR 3.61, 95% CI 2.18–6.00). Similarly, when omitting other studies, women with HDP were at higher risk for developing chronic hypertension than healthy controls. Sensitivity analysis of the PE group showed similar results after omitting other studies, and women with PE were at higher risk of developing chronic hypertension than those in the healthy control group (Figure 5B).

The quality assessment and risk of bias analysis of each included study are shown in Table 2.

### Meta-Regression Analysis

In the total pooled effect, the heterogeneity of the HDP group was *I*^2^ = 96%, and the heterogeneity of the PE group was *I*^2^ = 97%. Thus, we conducted meta-regression analysis based on the publication year, NOS score, country, sample size and study design. The results confirmed that the publication year and study design had a significant effect on the heterogeneity in the HDP group (*P*_publication year_ = 0.03, *P*_study design_ = 0.003). Other factors showed no significant effect on the heterogeneity in the HDP group. The publication year and study design may be the sources of heterogeneity for the experimental results. None of the factors showed a significant effect on the heterogeneity in the PE group (Table 3).

**TABLE 3:** Results of the meta-regression analysis.

Study level variables	HDP Group		PE Group	
	Coefficient (95% CI)	*P*-value	Coefficient (95% CI)	*P*-value
Publication year	(0.0077, 0.2026)	0.03	(−0.07, 0.16)	0.41
NOS score	(−1.28, 1.35)	0.96	(−3.10, 0.61)	0.19
Region	(−0.22, 0.82)	0.26	(−1.03, 1.28)	0.83
Sample size	(−1.31, 0.76)	0.6	(−1.78, 1.09)	0.64
Study design	(0.24, 1.17)	0.003	(−1.55, 2.19)	0.74

### Subgroup Analysis

We conducted subgroup analyses based on the year of publication (< 2016, ≥ 2016), study design, region (North America, South America, Europe, etc.), sample size (< 500, ≥ 500) and NOS score (< 7, ≥ 7) to further evaluate the correlations between HDP, GH, and PE and the risk of chronic hypertension. The subgroup analyses showed some inconsistencies; some of them seemed reasonable, while others did not.

**FIGURE 6:**
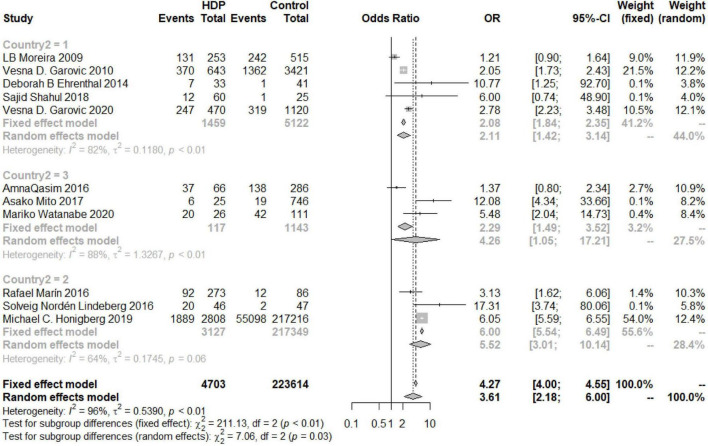
Subgroup analysis by region of the risk of developing chronic hypertension in the HDP group (1 = North and South America, 2 = Europe, 3 = Asia).

**FIGURE 7:**
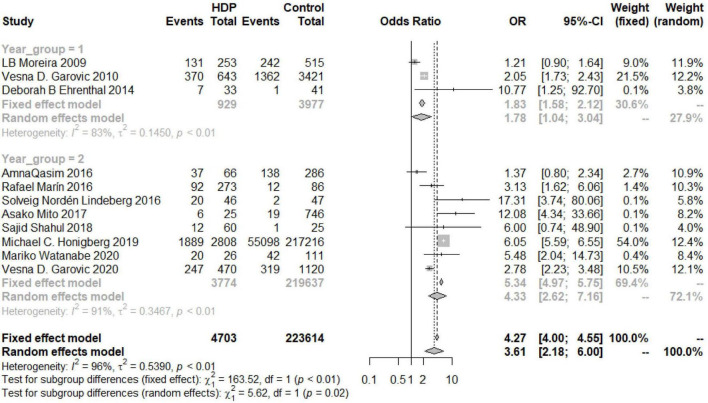
Subgroup analysis by publication year of the risk of developing chronic hypertension in the HDP group (Group 1 = years < 2016, Group 2 = years ≥ 2016).

**FIGURE 8:**
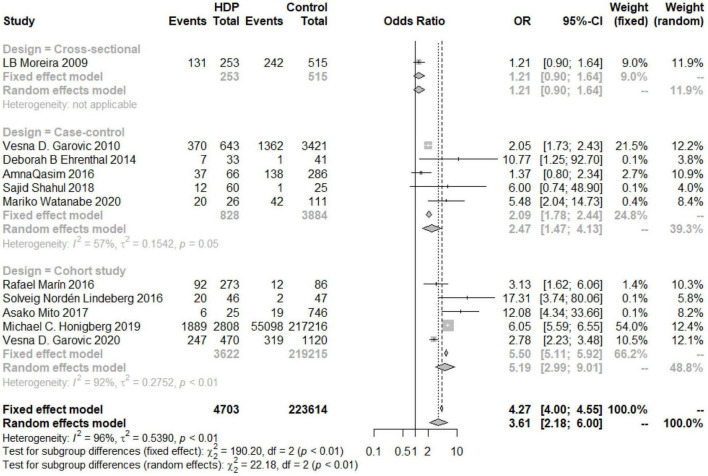
Subgroup analysis by the study design of the risk of developing chronic hypertension in the HDP group.

**FIGURE 9:**
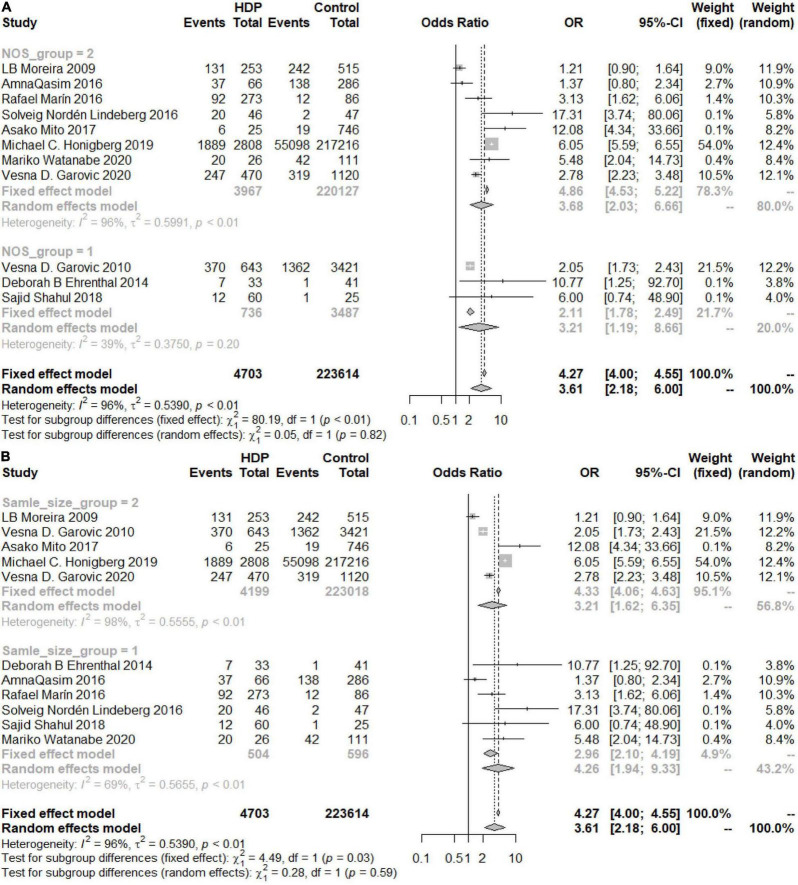
**(A)** Subgroup analysis by NOS score of the risk of developing chronic hypertension in the HDP group (Group 1 = NOS < 7, Group 2 = NOS ≥ 7). **(B)** Subgroup analysis by the sample size of the risk of developing chronic hypertension in the HDP group (Group 1 = sample size < 500, Group 2 = sample size ≥ 500).

An overall OR value of 5.75 (95% CI 3.92–8.44; *I*^2^ = 49%) was found for the risk of developing postpartum hypertension among women with a history of HDP. According to the subgroup analysis, the risk of chronic hypertension in patients with HDP increased for different continents, but there were differences among the continents (*P* = 0.03). The increased risk in North and South America was the lowest (OR 2.11, 95% CI 1.42–3.14), and the risk in Europe was the highest (OR 5.52, 95% CI 3.01–10.14), while the risk in Asia was similar to the overall assessment (OR 4.26, 95% CI 1.05–17.21) (Figure 6). According to the analysis of publication years, when the publication year was before 2016, the increase in the risk of developing chronic hypertension among patients with HDP was significantly lower than that among patients included in studies with a publication year of 2016 or later (*P* = 0.02, OR_<2016_ 1.78, 95% CI_<2016_ 1.04–3.04, OR_≥2016_ 4.33, 95% CI_≥2016_ 2.62–7.16) (Figure 7). Grouped by study design, the OR value of the case–control group was 2.47 (95% CI 1.47–4.13), that of the cohort control group was 5.19 (95% CI 2.99–9.01), and that of the cross-sectional group was 1.21 (95% CI 0.90–1.64) (Figure 8). According to the NOS score and sample size, the increased risk of developing chronic hypertension among HDP patients was similar to that of the overall evaluation (OR_NOS≥7_ 3.68, 95% CI_NOS≥7_ 2.03–6.66; OR_NOS<7_ 3.21, 95% _CINOS<7_ 1.19–8.66; OR_=500_ 3.21, 95% CI_=500_ 1.62–6.35; OR_≤500_ 4.26, 95% CI_≤500_ 1.94–9.33) (Figure 9).

**FIGURE 10:**
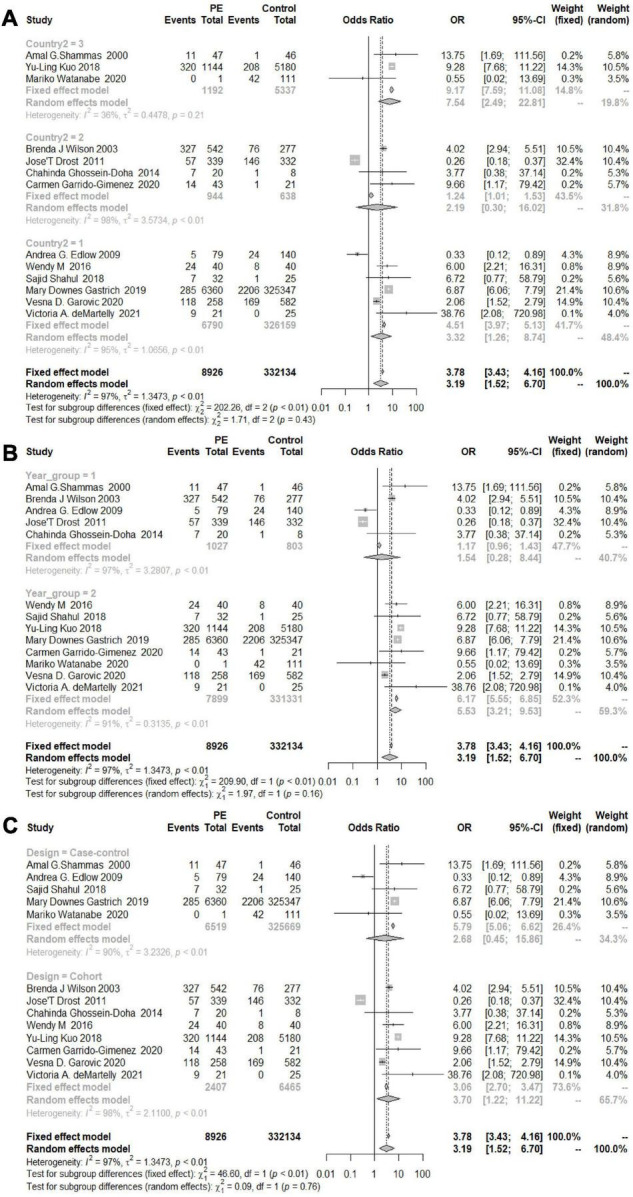
**(A)** Subgroup analysis by region of the risk of developing chronic hypertension in the PE group (1 = North and South America, 2 = Europe, 3 = Asia). **(B)** Subgroup analysis by publication year of the risk of developing chronic hypertension in the PE group (Group 1 = years < 2016, Group 2 = years ≥ 2016). **(C)** Subgroup analysis by the study design of the risk of developing chronic hypertension in the PE group.

**FIGURE 11:**
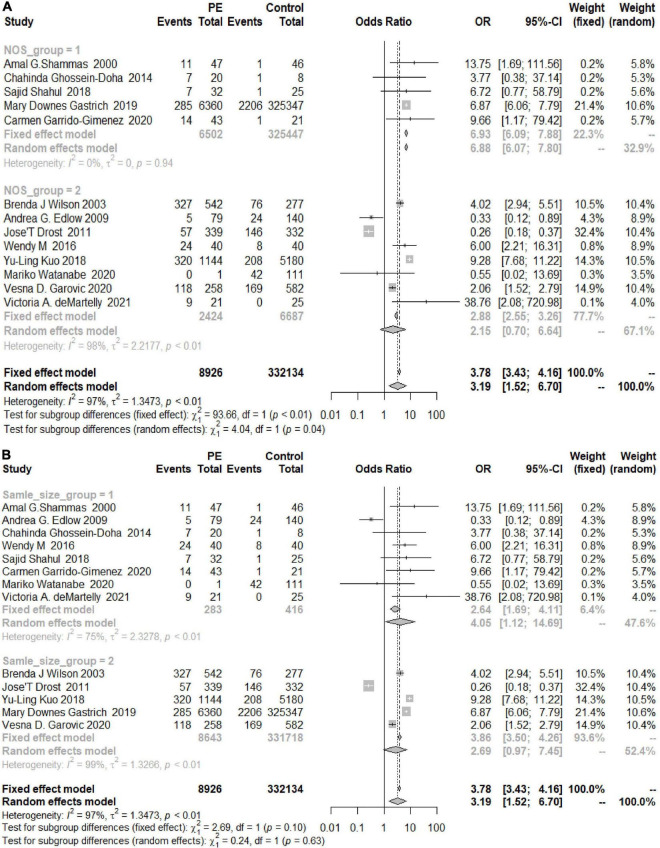
**(A)** Subgroup analysis by NOS score of the risk of developing chronic hypertension in the PE group (Group 1 = NOS < 7, Group 2 = NOS ≥ 7). **(B)** Subgroup analysis by sample size of the risk of developing chronic hypertension in the PE group (Group 1 = sample size < 500, Group 2 = sample size ≥ 500).

The overall OR was 3.19 (95% CI 1.52–6.70; *I*_2_ = 97%), and women with a history of PE had a greater risk of developing postpartum hypertension than women without PE. The increased risks in the Americas and Europe were similar to the overall risk (OR_Americas_ 3.32, 95% CI_America_ 1.26–8.74; OR_Europe_ 2.19, 95% CI_Europe_ 0.3–16.02), while the risk of developing chronic hypertension in Asia was significantly increased (OR 7.54, 95% CI 2.49–22.81) (Figure 10A). According to the analysis of the publication years, when the publication year was before 2016, the increase in the risk of developing chronic hypertension among patients with PE was significantly lower than that among patients included in studies with a publication year of 2016 or later (OR_<2016_ 1.54, 95% CI_<2016_ 0.28–8.44, OR_≥2016_ 5.53, 95% CI_≥2016_ 3.21–9.53) (Figure 10B). Grouped by study design, the OR value of the case–control group was 2.68 (95% CI 0.45, 15.86) and that of the cohort control group was 2.70 (95% CI 1.22, 11.22) (Figure 10C). The OR value of the NOS score ≥ 7 group was 2.15 (95% CI 0.7–6.64), and the OR of the other group was 6.88 (95% CI 6.07–7.80) (Figure 11A). According to sample size, the increase in the risk of developing chronic hypertension among PE patients was similar to that of the overall evaluation (OR_<500_ 4.05, 95% CI_<500_ 1.12–14.69; OR_≥500_ 2.69, 95%CI_≥500_ 0.97, 7.45) (Figure 11B).

## Discussion

### Principal Findings

**FIGURE 12:**
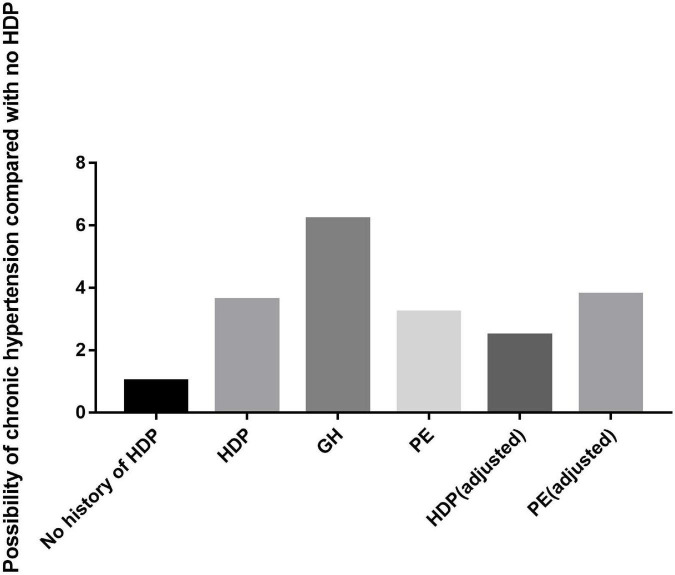
Main findings of the study.

Our systematic review and meta-analysis comprehensively explored the associations of HDP, GH, and PE with chronic hypertension. We included 21 articles with a total of 634,293 patients. The results of this systematic review and meta-analysis suggested that women with a history of HDP are almost 3.6 times more likely to develop chronic hypertension than those without a history of HDP, women with a history of GH are almost 6.2 times more likely to develop chronic hypertension than those without a history of GH, and women with a history of PE are almost 3.2 times more likely to develop chronic hypertension than those without a history of PE. In addition, we further calculated the probability of developing chronic hypertension among patients with HDP or PE after adjusting for age and BMI at recruitment, prepregnancy BMI, age at first delivery and other factors. The results suggested that women with a history of HDP were almost 2.47 times more likely to develop chronic hypertension than those without a history of HDP and that women with a history of PE were almost 3.78 times more likely to develop chronic hypertension than those without a history of PE (Figure 12). The above results show that women with HDP are more likely to develop chronic hypertension and that those with GH are more likely to have PE. Therefore, patients with HDP should monitor their blood pressure more actively in the future and choose a healthy lifestyle, such as a low-salt and low-fat diet, to reduce the possibility of hypertension. One meta-analysis showed that subclinical hypothyroidism during pregnancy is associated with an increased risk of developing HDP, and this association is present regardless of the gestational period (42). Some studies have shown that BMI or maternal prepregnancy obesity and abnormal gestational glucose metabolism are independently associated with an increased risk of HDP. Controlling these factors may reduce the occurrence of HDP (43, 44). Preventing or reducing the occurrence of HDP in pregnant women will inevitably reduce the probability of developing hypertension in the future. In terms of countries, women in Asian countries are more likely to develop chronic hypertension after HDP or PE, while the relative risk in the Americans is not high. This may be related to race, medical level and economic conditions. We look forward to future research.

### Comparison With Other Studies

Our systematic review illustrates the risk of developing chronic hypertension among pregnant women with HDP, GH and PE. Although the evidence linking pregnancy-induced hypertension with the development of hypertension has been recognized, there are still many outstanding problems in a number of specific aspects (45).

In 2007, a systematic review and meta-analysis showed that preeclampsia patients had more than three times the risk of developing hypertension (OR 3.70, 95% CI 2.70–5.05) than those without preeclampsia; the follow-up time was adjusted to 14.1 years (46). Subsequent studies did not adjust the follow-up years. A systematic review and meta-analysis in 2013 showed that women with a history of preeclampsia or eclampsia had more than three times the risk of developing hypertension (RR 3.13, 95% CI 2.51, 3.89) (14) than those without a history of preeclampsia or eclampsia. In 2016, Mayri Sagady Leslie reviewed 48 unique studies from 20 countries that included a total of 3,598,601 women, and found similar results (47). This outcome was consistent with ours. In 2018, L Brouwers’ team found that recurrent preeclampsia was consistently associated with an increased pooled risk ratio for hypertension (RR 2.3; 95% CI 1.9–2.9) (48). The above articles all studied the relationship between preeclampsia and chronic hypertension, and few meta-analyses have directly studied the relationship between HDP or GH and chronic hypertension.

The advantage of our study is that a large number of articles were selected, and the sample size was large. We not only studied the possibility of HDP leading to chronic hypertension but also accounted for the relevant data on various types of HDP and finally chose to analyze the large amount of relevant data for PE and GH. We also performed subgroup analysis (publication year, study design, country, sample size and NOS score) to analyze the sources of heterogeneity and the probability of developing chronic hypertension in each subgroup. In addition, we further calculated the probability of developing chronic hypertension for patients with HDP or PE after adjusting for age and BMI at recruitment, prepregnancy BMI, age at first delivery and other factors. In general, we carried out statistical analysis on all aspects of the obtained data that could be analyzed.

However, there are still some limitations of this study, which need further study. There are few studies with high scores. The ages of patients with HDP and chronic hypertension were not statistically analyzed because the data were seriously lacking, which may be the reason for the high heterogeneity. The published literature is insufficient to determine the best screening period for postpartum detection of hypertension. We could not determine an observation age or follow-up period to limit the screening of the articles. The heterogeneity of the population and hypertension definitions and the failure to obtain sufficient details make the results of the meta-analysis misleading, and they could not be adjusted using statistical tests.

## Conclusion

HDP, GH, and PE increase the likelihood that patients will develop chronic hypertension. After adjustment for age and BMI at recruitment, prepregnancy BMI, age at first delivery and other factors, patients with HDP or PE were still more likely to develop chronic hypertension. HDP, GH, and PE may be risk factors for chronic hypertension, independent of other risk factors.

## Data Availability Statement

The original contributions presented in this study are included in the article/supplementary material, further inquiries can be directed to the corresponding author/s.

## Author Contributions

JX, TL, and YW: study design, data extraction, statistical analysis, and manuscript writing. LX, ZM, WL, and KX: study design, data extraction, and verification. CH and HD: study design, statistical analysis, manuscript editing and reviewing, and funding. All authors contributed to the article and approved the submitted version.

## Conflict of Interest

The authors declare that the research was conducted in the absence of any commercial or financial relationships that could be construed as a potential conflict of interest.

## Publisher’s Note

All claims expressed in this article are solely those of the authors and do not necessarily represent those of their affiliated organizations, or those of the publisher, the editors and the reviewers. Any product that may be evaluated in this article, or claim that may be made by its manufacturer, is not guaranteed or endorsed by the publisher.

## References

[1] Sibai B Dekker G Kupferminc M. Pre-eclampsia. *Lancet.* (2005) 365:785–99. 10.1016/S0140-6736(05)71003-5 15733721

[2] Garovic VD Hayman SR. Hypertension in pregnancy: an emerging risk factor for cardiovascular disease. *Nat Clin Pract Nephrol.* (2007) 3:613–22. 10.1038/ncpneph0623 17957198

[3] Malek AM Hunt KJ Turan TN Mateus J. Hypertensive disorders of pregnancy with and without prepregnancy hypertension are associated with incident maternal kidney disease subsequent to delivery. *Hypertension.* (2022) 79:844–54. 10.1161/HYPERTENSIONAHA.121.18451 35209727 PMC8917087

[4] Bushnell C McCullough LD Awad IA Chireau MV Fedder WN Furie KL Guidelines for the prevention of stroke in women: a statement for healthcare professionals from the American Heart Association/American Stroke Association. *Stroke.* (2014) 45:1545–88. 10.1161/01.str.0000442009.06663.48 24503673 PMC10152977

[5] Mosca L Benjamin EJ Berra K Bezanson JL Dolor RJ Lloyd-Jones DM Effectiveness-based guidelines for the prevention of cardiovascular disease in women—2011 update. *J Am Coll Cardiol.* (2011) 57:1404–23. 10.1016/j.jacc.2011.02.005 21388771 PMC3124072

[6] Ananth CV Keyes KM Wapner RJ. Preeclampsia rates in the United States, 1980-2010:age-period-cohort analysis. *BMJ.* (2013) 347:f6564. 10.1136/bmj.f6564 24201165 PMC3898425

[7] Kuklina EV Ayala C Callaghan WM. Hypertensive disorders and severe obstetric morbidity in the United States. *Obstet Gynecol.* (2009) 113:1299–306. 10.1097/AOG.0b013e3181a45b25 19461426

[8] Cortés YI Catov JM Brooks M. Pregnancy-related events associated with subclinical cardiovascular disease burden in late midlife: swan. *Atherosclerosis.* (2019) 289:27–35. 10.1016/j.atherosclerosis.2019.07.012 31446211 PMC6952268

[9] Steinthorsdottir V McGinnis R Williams NO. Genetic predisposition to hypertension is associated with preeclampsia in European and Central Asian women. *Nat Commun.* (2020) 11:5976. 10.1038/s41467-020-19733-6 33239696 PMC7688949

[10] Parikh NI Gonzalez JM Anderson CAM. Adverse pregnancy outcomes and cardiovascular disease risk: unique opportunities for cardiovascular disease prevention in women: a scientific statement from the American Heart Association. *Circulation.* (2021) 143:e902–16. 10.1161/CIR.0000000000000961 33779213

[11] Svensson A Andersch B Hansson L. Prediction of later hypertension following a hypertensive pregnancy. *J Hypertens Suppl.* (1983) 1:94–6. 6599506

[12] Lindeberg S Axelsson O Jorner U Malmberg L Sandström B. A prospective controlled five-year follow up study of primiparas with gestational hypertension. *Acta Obstet Gynecol Scand.* (1988) 67:605–9. 10.3109/00016348809004272 3247832

[13] McDonald SD Malinowski A Zhou Q Yusuf S Devereaux PJ. Cardiovascular sequelae of preeclampsia/eclampsia: a systematic review and meta-analyses. *Am Heart J.* (2008) 156:918–30. 10.1016/j.ahj.2008.06.042 19061708

[14] Brown MC Best KE Pearce MS Waugh J Robson SC Bell R. Cardiovascular disease risk in women with pre-eclampsia: systematic review and meta-analysis. *Eur J Epidemiol.* (2013) 28:1–19. 10.1007/s10654-013-9762-6 23397514

[15] Wu P Haththotuwa R Kwok CS Babu A Kotronias RA Rushton C Preeclampsia and future cardiovascular health: a systematic review and meta-analysis. *Circ Cardiovasc Qual Outcomes.* (2017) 10:e003497. 10.1161/CIRCOUTCOMES.116.003497 28228456

[16] Carvalho MV Siqueira LB Sousa AL Jardim PC. The influence of hypertension on quality of life. *Arq Bras Cardiol.* (2013) 100:164–74. 10.5935/abc.20130030 23503826

[17] Gunderson EP Greenberg M Nguyen-Huynh MN. Early pregnancy blood pressure patterns identify risk of hypertensive disorders of pregnancy among racial and ethnic groups. *Hypertension.* (2022) 79:599–613. 10.1161/HYPERTENSIONAHA.121.18568 34963295 PMC9004135

[18] Moher D Liberati A Tetzlaff J Altman DG. Preferred reporting items for systematic reviews and meta-analyses: the PRISMA statement. *BMJ.* (2009) 339:b2535. 10.1136/bmj.b2535 19622551 PMC2714657

[19] Schokker SA Van Oostwaard MF Melman EM Van Kessel JP Baharoglu MI Roos YB Cerebrovascular, cardiovascular and renal hypertensive disease after hypertensive disorders of pregnancy. *Pregnancy Hypertens.* (2015) 5:287–93. 10.1016/j.preghy.2015.06.002 26597742

[20] Dormuth CR Filion KB Platt RW. Likelihood ratio meta-analysis: new motivation and approach for an old method. *Contemp Clin Trials.* (2016) 47:259–65. 10.1016/j.cct.2016.01.008 26837056 PMC5705233

[21] Garrido-Gimenez C Mendoza M Cruz-Lemini M Galian-Gay L Sanchez-Garcia O Granato C Angiogenic factors and long-term cardiovascular risk in women that developed preeclampsia during pregnancy. *Hypertension.* (2020) 76:1808–16. 10.1161/HYPERTENSIONAHA.120.15830 33012203

[22] Moreira LB Gus M Nunes G Gonçalves CB Martins J Wiehe M Association between pregnancy-related hypertension and severity of hypertension. *J Hum Hypertens.* (2009) 23:415–9. 10.1038/jhh.2008.140 19020534

[23] Drost JT Arpaci G Ottervanger JP de Boer MJ van Eyck J van der Schouw YT Cardiovascular risk factors in women 10 years post early preeclampsia: the Preeclampsia Risk EValuation in FEMales study (PREVFEM). *Eur J Prev Cardiol.* (2012) 19:1138–44. 10.1177/1741826711421079 21859777

[24] Watanabe M Sairenchi T Nishida K Uchiyama K Haruyama Y Satonaka H Gestational hypertension as risk factor of hypertension in middle-aged and older women. *Int J Environ Res Public Health.* (2020) 17:4052. 10.3390/ijerph17114052 32517151 PMC7312590

[25] Nordén Lindeberg S Hanson U. Hypertension and factors associated with metabolic syndrome at follow-up at 15 years in women with hypertensive disease during first pregnancy. *Hypertens Pregnancy.* (2000) 19:191–8. 10.1081/PRG-100100135 10877987

[26] Shammas AG Maayah JF. Hypertension and its relation to renal function 10 years after pregnancy complicated by pre-eclampsia and pregnancy induced hypertension. *Saudi Med J.* (2000) 21:190–2. 11533780

[27] Garovic VD Bailey KR Boerwinkle E Hunt SC Weder AB Curb D Hypertension in pregnancy as a risk factor for cardiovascular disease later in life. *J Hypertens.* (2010) 28:826–33. 10.1097/HJH.0b013e328335c29a 20087214 PMC2980863

[28] Wilson BJ Watson MS Prescott GJ Sunderland S Campbell DM Hannaford P Hypertensive diseases of pregnancy and risk of hypertension and stroke in later life: results from cohort study. *BMJ.* (2003) 326:845. 10.1136/bmj.326.7394.845 12702615 PMC153466

[29] Mito A Arata N Qiu D Sakamoto N Murashima A Ichihara A Hypertensive disorders of pregnancy: a strong risk factor for subsequent hypertension 5 years after delivery. *Hypertens Res.* (2018) 41:141–6. 10.1038/hr.2017.100 29093561

[30] Garovic VD White WM Vaughan L Saiki M Parashuram S Garcia-Valencia O Incidence and long-term outcomes of hypertensive disorders of pregnancy. *J Am Coll Cardiol.* (2020) 75:2323–34. 10.1016/j.jacc.2020.03.028 32381164 PMC7213062

[31] Edlow AG Srinivas SK Elovitz MA. Investigating the risk of hypertension shortly after pregnancies complicated by preeclampsia. *Am J Obstet Gynecol.* (2009) 200:e60–2. 10.1016/j.ajog.2008.10.012 19111719

[32] Honigberg MC Zekavat SM Aragam K Klarin D Bhatt DL Scott NS Long-term cardiovascular risk in women with hypertension during pregnancy. *J Am Coll Cardiol.* (2019) 74:2743–54. 10.1016/j.jacc.2019.09.052 31727424 PMC6981240

[33] Marín R Gorostidi M Portal CG Sánchez M Sánchez E Alvarez J. Long-term prognosis of hypertension in pregnancy. *Hypertens Pregnancy.* (2000) 19:199–209. 10.1081/PRG-100100136 10877988

[34] Kuo YL Chan TF Wu CY Ker CR Tu HP. Preeclampsia-eclampsia and future cardiovascular risk among women in Taiwan. *Taiwan J Obstet Gynecol.* (2018) 57:364–9. 10.1016/j.tjog.2018.04.035 29880166

[35] Gastrich MD Zinonos S Bachmann G Cosgrove NM Cabrera J Cheng JQ Preeclamptic women are at significantly higher risk of future cardiovascular outcomes over a 15-year period. *J Womens Health (Larchmt).* (2020) 29:74–83. 10.1089/jwh.2019.7671 31414929

[36] White WM Mielke MM Araoz PA Lahr BD Bailey KR Jayachandran M A history of preeclampsia is associated with a risk for coronary artery calcification 3 decades later. *Am J Obstet Gynecol.* (2016) 214:519-519.e1–8. 10.1016/j.ajog.2016.02.003 26874301 PMC4808608

[37] Qasim A Bashir A Sajid S Riaz MM Almas A. Women with pregnancy induced hypertension have a higher risk of developing essential hypertension - a case control study from a tertiary care center in Pakistan. *J Pak Med Assoc.* (2016) 66:179–83. 26819164

[38] Ghossein-Doha C Spaanderman M van Kuijk SM Kroon AA Delhaas T Peeters L. Long-term risk to develop hypertension in women with former preeclampsia: a longitudinal pilot study. *Reprod Sci.* (2014) 21:846–53. 10.1177/1933719113518989 24440998 PMC4107566

[39] Ehrenthal DB Goldstein ND Wu P Rogers S Townsend RR Edwards DG. Arterial stiffness and wave reflection 1 year after a pregnancy complicated by hypertension. *J Clin Hypertens (Greenwich).* (2014) 16:695–9. 10.1111/jch.12398 25116457 PMC4192066

[40] Shahul S Ramadan H Nizamuddin J Mueller A Patel V Dreixler J Activin A and late postpartum cardiac dysfunction among women with hypertensive disorders of pregnancy. *Hypertension.* (2018) 72:188–93. 10.1161/HYPERTENSIONAHA.118.10888 29844146

[41] Martelly VA Dreixler J Tung A Mueller A Heimberger S Fazal AA Long-term postpartum cardiac function and its association with preeclampsia. *J Am Heart Assoc.* (2021) 10:e018526. 10.1161/JAHA.120.018526 33619970 PMC8174300

[42] Han Y Wang J Wang X. Relationship between subclinical hypothyroidism in pregnancy and hypertensive disorder of pregnancy: a systematic review and meta-analysis. *Front Endocrinol (Lausanne).* (2022) 13:823710. 10.3389/fendo.2022.823710 35355565 PMC8959212

[43] Honigberg MC Chaffin M Aragam K. Genetic variation in cardiometabolic traits and medication targets and the risk of hypertensive disorders of pregnancy. *Circulation.* (2020) 142:711–3. 10.1161/CIRCULATIONAHA.120.047936 32804569 PMC7436942

[44] Li MF Ke JF Ma L Wang JW. Maternal pre-pregnancy obesity combined with abnormal glucose metabolism further increases adverse pregnancy outcomes in Chinese pregnant women. *Front Endocrinol (Lausanne).* (2022) 12:754406. 10.3389/fendo.2021.754406 35095754 PMC8793842

[45] Hauspurg A Countouris ME Catov JM. Hypertensive disorders of pregnancy and future maternal health: how can the evidence guide postpartum management? *Curr Hypertens Rep.* (2019) 21:96. 10.1007/s11906-019-0999-7 31776692 PMC7288250

[46] Bellamy L Casas JP Hingorani AD Williams DJ. Pre-eclampsia and risk of cardiovascular disease and cancer in later life: systematic review and meta-analysis. *BMJ.* (2007) 335:974. 10.1136/bmj.39335.385301.BE 17975258 PMC2072042

[47] Leslie MS Briggs LA. Preeclampsia and the risk of future vascular disease and mortality: a review. *J Midwifery Womens Health.* (2016) 61:315–24. 10.1111/jmwh.12469 27155218

[48] Brouwers L van der Meiden-van Roest AJ Savelkoul C Vogelvang TE Lely AT Franx A Recurrence of pre-eclampsia and the risk of future hypertension and cardiovascular disease: a systematic review and meta-analysis. *BJOG.* (2018) 125:1642–54. 10.1111/1471-0528.15394 29978553 PMC6283049

